# The “enthesis organ” concept and its relevance to foot and ankle pathology: a literature review

**DOI:** 10.1186/1757-1146-4-S1-P56

**Published:** 2011-05-20

**Authors:** Tom Walsh, Chris Bishop

**Affiliations:** 1 Podiatry Department, Repatriation General Hospital, Daws Park, SA, Australia

## Background

An “enthesis’ is described as a junction between tendon, ligament or joint capsule and bone. Tendinous entheses are a complex and a particularly important entity, as they are responsible for ensuring contractile force generated by muscle is transmitted to the skeleton. While complaints of pain at entheses sites are common, rupture or avulsion of tendinous tissue is not. This review aims to provide insight into what mechanisms the musculoskeletal system has developed for protecting tendinous entheses and what are the effects when these mechanisms are compromised (enthesopathy).

## Method

An electronic database search was performed in October 2010. The search strategy used was enthesis* AND foot* AND (ankle* OR enthesopathy* OR enthesis organ* OR enthesitis). A secondary snowball method was conducted targeting reference lists and literature only available in hard copy.

## Results

The initial electronic database search identified 526 articles. 468 were excluded based on title and abstract alone. Inclusion and exclusion criteria were applied and excluded a further 26 articles. The snow ball method retrieved a further 7 articles. 39 articles were included in the review. The literature identified comprehensive investigations on anatomical, functional and pathophysiological details of tendinous entheses at varied locations of the foot an ankle. On histological examination, there is extensive interweaving of tendinous tissue with fibrocartilage and bone to form the true enthesis. This interweaving leaves the attachment incredibly robust. Histological sections also revealed structures adjacent, and in close apposition, to entheses that would appear to reduce tensile, compressive and shear stress applied during muscular contraction. The arrangement of these structures has been termed the "enthesis organ". Enthesopathy was also found to be closely linked with spondyloarthropathies.

## Conclusion

Pathology of the entheses, enthesopathy, can develop for various reasons including post traumatic or rheumatological. The signs and symptoms of enthesopathy can be varied and can include formation of enthesophytes with chronic pain to changes in nail structure The review describes the associated anatomy of entheses, the process of enthesopathy and its association with spondyloarthropathies. It will introduce and consolidate podiatrists’ understanding of enthesopathies and how they present in the foot and ankle.

